# Developmental and Structural Alterations at the Ductus–Aortic Isthmus Interface in Infantile Coarctation of the Aorta: A Biological Basis for Persistent Vascular Disease Beyond Anatomical Repair

**DOI:** 10.3390/jcm15135214

**Published:** 2026-07-03

**Authors:** Isabell G. Robl, Robert Cesnjevar, Arif B. Ekici, Steffen Uebe, Pascal D. Johann, Maria Daniela Hernandez Ramirez, Victoria E. Fincke, Fabian B. Fahlbusch, Julia Moosmann

**Affiliations:** 1 Neonatology and Pediatric Intensive Care, Faculty of Medicine, University of Augsburg, Stenglinstr. 2, 86156 Augsburg, Germany; isabellgisela.robl@uk-augsburg.de; 2 Department of Heart Surgery, Friedrich-Alexander-Universität Erlangen-Nürnberg (FAU), Krankenhausstraße 12, 91054 Erlangen, Germany; 3 Institute of Human Genetics, Friedrich-Alexander-Universität Erlangen-Nürnberg (FAU), Schwabachanlage 10, 91054 Erlangen, Germany; 4 Pediatric and Adolescent Medicine, Swabian Children’s Cancer Center Augsburg, EU-RHAB Trial Center, Germany and Bavarian Cancer Research Center (BZKF), 86156 Augsburg, Germany; 5 Department of Pediatric Cardiology, Charité-Universitätsmedizin Berlin, Campus Virchow-Klinikum, Augustenburger Platz 1, 13353 Berlin, Germany; julia.moosmann@dhzc-charite.de

**Keywords:** coarctation of the aorta, aortic isthmus, developmental patterning, extracellular matrix remodeling, *HOX* genes, retinoic acid signaling, *TFAP2B*, vascular smooth muscle, transcriptomics, Ingenuity Pathway Analysis

## Abstract

**Background:** Coarctation of the aorta (CoA) is a congenital narrowing of the aortic isthmus near the ductus arteriosus or ligamentum arteriosum. Despite successful anatomical repair, patients remain at risk of recoarctation, arterial hypertension, and diffuse aortopathy, suggesting intrinsic vessel-wall abnormalities beyond localized obstruction. The developmental and molecular basis of these persistent vascular features remains incompletely understood. **Methods:** Human aortic tissue samples were obtained from 8 male infants with CoA and 6 age- and sex-matched controls aged <1 year. Total RNA was isolated, and gene expression profiling was performed using whole human genome oligo microarrays (Agilent). Differentially expressed transcripts were subjected to pathway, network, and upstream regulator analyses using Ingenuity Pathway Analysis (IPA, Qiagen). Selected candidate genes were evaluated by RT-qPCR in independent verification sets. **Results:** Transcriptomic profiling identified 402 analysis-ready transcripts distinguishing CoA from control tissue. Exploratory pathway analyses suggested extracellular matrix remodeling characterized by collagen turnover, integrin-mediated cell–matrix interactions, wound-healing signaling, and fibrosis-associated programs. In addition, enrichment analyses identified developmental annotations involving retinoic acid (RA)/RAR/RXR signaling, *HOX*-associated developmental programs, and a shared *HOX/MEIS*-associated signature. Network and upstream regulator analyses further suggested associations with cytoskeletal, muscle-associated, and epigenetic regulatory pathways, including *KAT6A*, *KAT6B*, retinoic acid/RAR/RXR signaling, *DNMT3B*, *KMT2A*, and *ARID1A.* RT-qPCR independently confirmed increased expression of *EDN1*, *AGTR2*, *IRS4*, and *TFAP2B.* **Conclusions:** Infantile CoA tissue exhibited molecular signatures consistent with vessel-wall remodeling accompanied by developmental, vascular signaling, and smooth muscle/cytoskeletal regulatory programs. These findings support the hypothesis that developmental patterning signals and postnatal extracellular matrix remodeling coexist within CoA tissue and may contribute to persistent vascular abnormalities beyond anatomical repair. Given the exploratory nature of the study, these observations should be considered hypothesis-generating and require validation in independent cohorts.

## 1. Introduction

Coarctation of the aorta (CoA) accounts for approximately 4–8% of congenital heart defects and is more common in males than in females [1,2,3]. It is characterized by narrowing of the aorta, typically at the aortic isthmus near the ductus arteriosus or ligamentum arteriosum, and frequently occurs together with other cardiovascular anomalies, including bicuspid aortic valve, septal defects, aortic arch hypoplasia, and patent ductus arteriosus (PDA) [3,4]. Familial clustering, syndromic associations such as Turner or Kabuki syndrome, and emerging data on single-gene variants and copy number changes support a genetic contribution, although the molecular mechanisms underlying isolated CoA remain incompletely understood [5,6,7,8].

The standard of treatment in neonates and infants is surgical repair, which achieves good early survival, most commonly through resection of the stenotic segment with end-to-end or extended end-to-end anastomosis [9,10]. However, CoA remains associated with substantial lifelong morbidity, including recoarctation, arterial hypertension, reduced arterial compliance, and diffuse aortopathy despite anatomically successful repair [11,12,13]. These sequelae suggest that CoA is not solely a discrete mechanical obstruction, but may also reflect intrinsic abnormalities of aortic wall biology, regional vessel identity, and postnatal remodeling.

Several embryologic mechanisms have been proposed to explain CoA formation, including constriction of ectopic ductal tissue within the aortic wall, altered fetal hemodynamics affecting aortic arch and isthmic growth, and abnormal remodeling or involution of the left dorsal aorta during early vascular development [14,15,16,17].

These mechanisms are not mutually exclusive and converge anatomically in the aortic arch–ductus–isthmus region. Development of this region depends on coordinated embryonic patterning, pharyngeal arch artery remodeling, neural crest cell migration, endothelial differentiation, and vascular smooth muscle cell specification [18]. Cardiac and cranial neural crest cells contribute to pharyngeal arch derivatives, outflow tract and great-vessel patterning, and components of the aortic arch, making neural crest-associated developmental programs biologically relevant candidates for involvement in CoA pathogenesis [19,20,21,22,23].

At the same time, regional vascular identity is shaped by broader positional and epigenetic patterning programs. Retinoic acid/RAR–RXR signaling [24], *HOX* cofactors such as *MEIS* and *PBX* [25], and chromatin regulators [26] participate in establishing and maintaining such positional programs. *HOX* transcription factors define anterior–posterior identity during embryogenesis and may contribute to regionally restricted expression beyond embryonic development [27,28]. Emerging evidence suggests that such developmental programs may influence postnatal vascular phenotype, including vascular smooth muscle cell biology, extracellular matrix (ECM) organization, vascular remodeling, and inflammatory vascular disease [29,30]. Thus, CoA may arise at the intersection of altered embryonic positional identity and subsequent vessel-wall remodeling rather than from a single isolated developmental defect.

Molecular data from localized human CoA tissue remain limited. Recent transcriptomic work has highlighted associations between age, sex, ECM accumulation, fibrosis-related signaling, and inflammatory activation within the coarctation area [14]. These findings emphasize the need for carefully controlled analyses of infantile CoA tissue that distinguish early developmental and remodeling-related signals from age- and sex-dependent downstream processes.

In the present study, we analyzed localized CoA tissue from male infants and age- and sex-matched external vascular controls to reduce biological heterogeneity.

We aimed to determine whether infantile CoA tissue exhibits molecular signatures consistent with developmental patterning processes at the aortic arch–ductus–isthmus interface and whether such signatures coexist with ECM, smooth muscle, and vascular remodeling programs.

We hypothesized that infantile CoA may represent a developmental-remodeling lesion in which RA/*HOX*-associated developmental signatures, neural crest- and pharyngeal arch-associated developmental programs, and postnatal vessel-wall remodeling processes coexist within the affected tissue. This developmental perspective is clinically relevant because CoA treatment is not limited to relieving a fixed anatomical obstruction; even after successful surgical repair, recoarctation, residual arch obstruction, and arterial hypertension may occur, suggesting that underlying vessel-wall and developmental programs could influence long-term treatment trajectories.

## 2. Materials and Methods

### 2.1. Patients and Tissue Sampling

**Figure 1:**
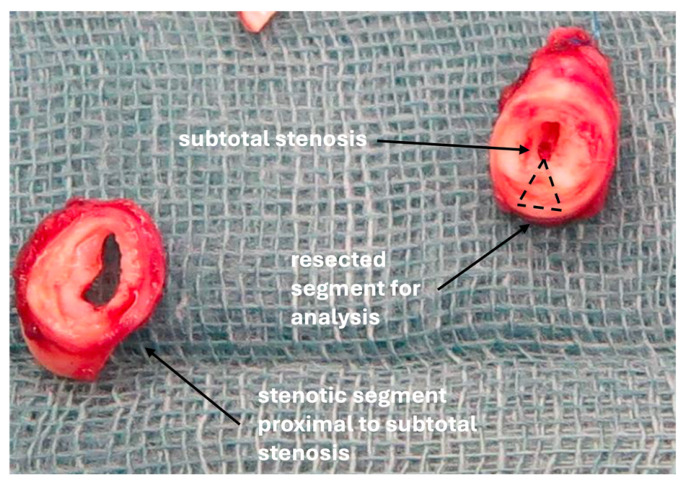
Representative intraoperative sampling of coarctation tissue. The image illustrates the stenotic aortic segment selected for molecular analysis. The left panel shows the segment proximal to the subtotal stenosis, and the right panel shows the subtotal stenotic region. The triangle indicates the resected tissue segment used for molecular analyses.

This retrospective, biobank-based study used archived surgically resected aortic tissue collected between 2011 and 2013 through the biobank infrastructure of the Competence Network for Congenital Heart Defects, Germany, and the National Register for Congenital Heart Defects, Germany. For microarray-based transcriptome analysis, aortic tissue samples were available from 6 control patients (Ao) and 8 patients with CoA (Table 1). To reduce biological heterogeneity and account for potential sex- and age-related effects, only male infants younger than one year were included. Owing to ethical and practical limitations precluding the acquisition of healthy infant aortic tissue, control samples were derived from patients undergoing surgery for other congenital cardiovascular malformations. The majority of control cases consisted of dextro-transposition of the great arteries (d-TGA; n = 4), supplemented by one case of pulmonary atresia with ventricular septal defect and major aortopulmonary collateral arteries (MAPCAs; n = 1) and one case of subvalvular pulmonary stenosis (n = 1). Control tissue was obtained from macroscopically normal-appearing aortic tissue collected during these surgical procedures. In contrast, the CoA cohort exhibited an isolated aortic phenotype without concomitant structural cardiovascular abnormalities, and CoA samples were obtained from the surgically resected stenotic aortic isthmus segment during corrective repair (Figure 1).

### 2.2. RNA Extraction

Aortic tissue samples were cryoconserved after surgical collection and subsequently processed according to the requirements of the respective downstream analyses. For transcriptome profiling, RNA extraction from dry-frozen aortic tissue samples was performed by AROS Applied Biotechnology A/S (Aarhus, Denmark) using the RNeasy Fibrous Tissue Mini Kit (Qiagen GmbH, Düsseldorf, Germany). For RT-qPCR verification, independent cDNA sets from CoA and ascending aortic (Ao) tissue samples were obtained through the biobank infrastructure of the Competence Network for Congenital Heart Defects and the National Register for Congenital Heart Defects, as described above.

### 2.3. Transcriptome Analysis

RNA samples were further processed by Miltenyi Biotec GmbH (Bergisch Gladbach, Germany) using the Agilent Whole Human Genome Oligo Microarray platform (SurePrint G3 Human GE v2 8 × 60K Microarray, Agilent Technologies, Santa Clara, CA, USA). RNA amplification, labeling, hybridization, washing, and scanning were performed according to the manufacturer’s protocols for the Agilent platform; detailed experimental protocols are provided in the GEO submission. All samples included in the analysis showed sufficient RNA quality (RIN > 6 [31]; Agilent 2100 Bioanalyzer platform) and adequate hybridization results. Agilent Feature Extraction Software (v12.1, Agilent Technologies) was used for extraction of microarray signal intensities. Differential gene expression analysis between the two study groups was subsequently performed in R (version 4.4.1), as described below. The microarray data have been deposited in NCBI’s Gene Expression Omnibus (GEO) [32] and are accessible under GEO Series accession number GSE337213.

#### 2.3.1. Exploratory Dimensionality Reduction

Uniform Manifold Approximation and Projection (UMAP) was applied as an exploratory dimensionality reduction technique to visualize sample-level structure and clustering within the two groups, Ao and CoA. UMAP was run with 15 neighbors and a minimum distance of 0.1 to emphasize local relationships between samples.

#### 2.3.2. Hierarchical Clustering

Genes were ranked based on a combined metric of statistical significance and variability, prioritizing transcripts that were both differentially expressed and highly variable across samples. The top 1000 genes were selected for hierarchical clustering. Clustering was performed using correlation-based distances and average-linkage clustering. A heatmap was generated using the R package pheatmap (version 1.0.13; https://CRAN.R-project.org/package=pheatmap, accessed on 28 June 2026) to visualize gene expression patterns across samples. Log2-transformed expression data were used for visualization.

#### 2.3.3. Gene Expression Processing and Differential Expression Analysis

Gene expression data were analyzed in R using the limma package (version 3.60.6; DOI: 10.18129/B9.bioc.limma). Expression values corresponding to the selected male Ao and CoA samples were extracted and organized into an expression matrix. Expression intensities were log2-transformed following addition of a pseudocount of 1 to stabilize variance and facilitate comparative analysis between the two experimental groups, Ao controls and CoA samples. Probes with low expression were removed prior to downstream analysis, retaining only probes with log2-transformed expression values > 5 in at least 50% of samples. Differential gene expression analysis between Ao control and CoA samples was performed using linear models implemented in the limma package. A design matrix was constructed to define the two experimental groups, and contrasts were specified to compare CoA versus Ao samples. Empirical Bayes moderation was applied to stabilize variance estimates across probes and improve statistical robustness. For each probe, differential expression was quantified as the log2 fold change (logFC) between CoA and Ao samples. Positive logFC values indicate increased expression in CoA samples relative to Ao controls, whereas negative logFC values indicate decreased expression in CoA samples.

#### 2.3.4. Statistical Analysis

Statistical significance of differential gene expression was assessed using moderated *t*-statistics generated within the limma empirical Bayes framework. Resulting *p*-values were corrected for multiple hypothesis testing using the Benjamini–Hochberg false discovery rate (FDR) method. Given the rarity of available infant aortic tissue and the exploratory nature of this study, genes with an adjusted *p*-value (FDR) < 0.20 and an absolute log2 fold change (|logFC|) ≥ 0.5 were considered differentially expressed and retained for exploratory downstream analyses. For transparency, the numbers of transcripts remaining significant at FDR < 0.05 and FDR < 0.10 were additionally evaluated.

### 2.4. Functional Pathway Analysis

To investigate biological pathways, molecular networks, and inferred upstream regulators, we used QIAGEN Ingenuity Pathway Analysis (IPA, QIAGEN Inc., Redwood City, CA, USA; version 24.0.2, released May 2025), as previously described by us [33] and others [14]. IPA maps differentially represented genes to curated pathways, networks, disease/function annotations, and regulator–target relationships within the Ingenuity Knowledge Base.

The differential expression dataset was initially prefiltered using an FDR threshold of 0.2 and an absolute log2 fold-change threshold of ≥0.5. For downstream IPA, the analysis-ready subset was defined using a nominal *p*-value together with a stricter absolute log2 fold-change threshold of ≥1, corresponding to transcripts with log2FC ≤ −1 or ≥+1. Only molecules meeting this more stringent downstream criterion were included in the IPA input dataset. The resulting gene list was analyzed in an exploratory setting using nominal *p*-values and log_2_ fold-change values for pathway ranking and directional interpretation. Canonical pathways were ranked by −log(*p*-value), and activation z-scores were used, where available, to indicate predicted activation or inhibition. Experimentally observed relationships from human, mouse, and rat orthologs were included. IPA results were interpreted as hypothesis-generating; disease- or organ-specific pathway labels were interpreted as functional annotations based on shared molecular components rather than as evidence of organ-specific pathology.

### 2.5. Quantitative PCR

RT-qPCR using TaqMan probes was performed for relative mRNA quantitation of endothelin 1 and 2 (*EDN1*, *EDN2*), endothelin type A receptor (*EDNRA*), angiotensin II receptor types 1 and 2 (*AGTR1*, *AGTR2*), cardiac muscle alpha actin (*ACTC1*), transforming growth factor beta 1 (*TGFB1*), ADP-ribosylation factor 6 (*ARF6*), insulin receptor substrate 4 (*IRS4*), trefoil factor 3 (*TFF3*), and transcription factor AP-2 beta (*TFAP2B*) on the Applied Biosystems 7500 Real-Time PCR system (Thermo Fisher Scientific, Waltham, MA, USA). These genes were selected as representative candidate transcripts emerging from the transcriptomic and pathway analyses and were used for independent transcript-level verification. *ARF6*, *TGFB1*, *EDNRA*, *EDN1* and *AGTR1*, *2* mRNA was analyzed in subset one, while *ACTC1*, *EDN2*, *IRS4*, *TFF3*, and *TFAP2B* mRNA expression was determined in subset two (see above). One-step RT-PCR amplification was carried out in a total 25 μL reaction mixture consisting of 10× TaqMan buffer II, 2.5 mM MgCl_2_, ROX Passive, 5 mM dNTP-Mix (UTP), AmpliTaq Gold DNA Polymerase, AmpliTaq gold, AmpERaseUNg, H_2_O, 2.5 μL of each primer, and 2.5 μL probe. Thermal cycle conditions were 50 °C for 2 min and 95 °C for 10 min, followed by 40 cycles at 94 °C for 15 s and 60 °C for 1 min. All calculations were based on the ∆∆Ct-method. Expression was calculated against a standard curve with serial dilutions of total RNAs from aortic tissue and compared to three housekeeping genes (hypoxanthine phosphoribosyl transferase—*HPRT1*, glyceraldehyde 3-phosphate dehydrogenase—*GAPDH*, and betaactin—*ACTB*). Experiments were repeated twice for each sample. Results are presented as relative quantitation (RQ; normalized percent of control expression). Primers and Probes are listed in Supplementary Table S1. Statistical analysis for the TaqMan PCR experiments was performed using GraphPad Prism version 10.4.1 (GraphPad Software, San Diego, CA, USA). Continuous variables are presented as mean ± standard deviation (SD). Normality of data distribution was assessed by the Shapiro–Wilk test. Depending on distributional characteristics, Welch’s two-sided unpaired *t*-test was applied to evaluate differences in gene expression, as it does not assume equal variances and remains valid under heteroscedastic conditions. A *p*-value of <0.05 was considered statistically significant.

### 2.6. Institutional Review Board Statement and Informed Consent

This study was approved by the responsible institutional ethics committees of (i) Friedrich-Alexander-Universität (FAU) Erlangen-Nürnberg, Erlangen, Germany (approval no. 3818/Addendum, 12 May 2010), and (ii) Charité—Universitätsmedizin Berlin, Berlin, Germany (approval no. EA2/131/10, 18 April 2011). The latter constituted the complementary ethics approval governing biobanking and tissue collection at the Berlin site. Participant inclusion and tissue collection were conducted between 2011 and 2013 and commenced at each participating site only after approval had been granted by the respective responsible ethics committee. Written informed consent for participant inclusion, biobanking, and subsequent scientific use of the tissue samples was obtained from all legal guardians before inclusion. All procedures involving human tissue were performed in accordance with institutional protocols, established clinical standards, and relevant regulatory guidelines. The study was conducted in full compliance with the ethical principles outlined in the Declaration of Helsinki [34].

### 2.7. Generative AI and AI-Assisted Technologies in the Writing Process

During the preparation of this manuscript, the authors used AI-assisted language tools to improve readability and language quality. All generated content was critically reviewed, revised, and verified by the authors, who take full responsibility for the content of this publication.

## 3. Results

For functional pathway analysis via gene array, aortic tissue samples were obtained from 6 Ao and 8 CoA patients in their first year of life. The mean age ± standard deviation (SD) of Ao and CoA patients at the time of surgery was not significantly different, i.e., 122.83 ± 144.80 days (n = 6) and 34.88 ± 31.46 (n = 8, *p* = 0.2) days, respectively.

### 3.1. Global Transcriptomic Profile of Infantile CoA Tissue

Following preprocessing and filtering, 468 transcripts were identified in the initial differential-expression dataset. Of these, 402 transcripts fulfilled the predefined criteria for downstream pathway analysis. The filtering approach used an initial false discovery rate threshold of 0.2 and subsequent pathway-analysis input criteria based on nominal *p* values and an absolute log_2_ fold-change threshold of ≥1. The resulting analysis-ready gene set comprised 337 upregulated and 65 downregulated transcripts in CoA tissue compared with control vascular tissue. It was found that 213 transcripts remained significant at FDR < 0.05 and 318 transcripts at FDR < 0.10, indicating that a substantial proportion of the differential expression signal persisted under more stringent significance criteria.

**Figure 2:**
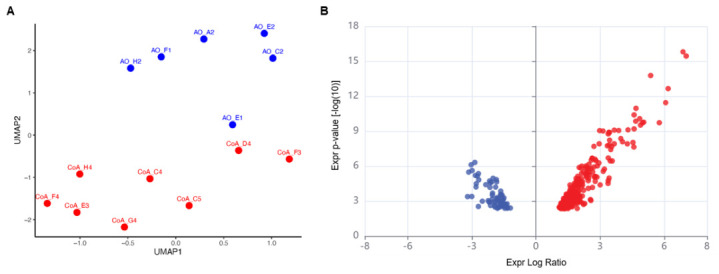
Transcriptomic group separation and differential transcript representation. (**A**) Exploratory Uniform Manifold Approximation and Projection (UMAP) analysis of CoA and Ao tissue samples. Legend: AO, ascending aortic control tissue; CoA, coarctation of the aorta. Blue dots indicate Ao controls; red dots indicate CoA samples. (**B**) Volcano plot of differentially expressed transcripts between CoA and Ao tissue generated using IPA. Legend: IPA, Ingenuity Pathway Analysis; Ao, ascending aortic control tissue; CoA, coarctation of the aorta. Blue dots indicate transcripts downregulated in CoA tissue relative to Ao controls; red dots indicate transcripts upregulated in CoA tissue relative to Ao controls.

Unsupervised dimensionality-reduction analyses indicated group-wise separation between CoA and Ao control samples. Uniform Manifold Approximation and Projection (UMAP) analysis demonstrated clustering according to tissue group (Figure 2A). A volcano plot (Figure 2B) generated from IPA software was used to visualize the distribution of differentially expressed transcripts and selected highly regulated genes. To provide gene-level context for the pathway-based analyses, the 15 most strongly up- and downregulated transcripts, ranked by log_2_ fold change, are listed in Table 2. Individual immune-related transcripts among the most strongly downregulated genes were interpreted cautiously and in the context of the broader pathway- and network-level results. This curated transcript set served as the input for canonical pathway analysis, network generation, and upstream regulator analysis.

### 3.2. Canoncal Pathway Analysis Highlights Extracellular Matrix Remodeling and Developmental Patterning Signatures

Canonical pathway analysis of the 402-transcript input set identified a predominant ECM and collagen-remodeling signature in CoA tissue. This signature was reflected by enrichment of several IPA pathways related to fibrosis-like tissue remodeling, collagen biosynthesis and maturation, collagen chain trimerization, collagen degradation, integrin-mediated cell–matrix interaction, wound-healing signaling, fibril assembly, and ECM organization. These pathways were driven by recurrent collagen and matrix-associated transcripts, including *COL11A2*, *COL13A1*, *COL16A1*, *COL4A5*, *COL4A6*, *COL6A6*, *COL8A1*, *COL8A2*, *COL9A1*, *P4HA3*, *PLOD2*, *LOXL2*, *MMP11*, *ITGA3*, *TNC*, *LAMC3*, *PDGFB*, and *PDGFRB*. The top 20 enriched canonical pathways are shown in Table 3. The full canonical pathway output with pathway-associated molecules is provided in Supplementary Table S2.

In addition to this dominant remodeling signature, canonical pathway analysis retained a distinct developmental patterning component. Among the enriched pathways were RAR activation and activation of anterior *HOX* genes in hindbrain during early embryogenesis, involving multiple *HOX* paralogs and developmental cofactors, including *HOXA3*, *HOXA4*, *HOXA5*, *HOXB2*, *HOXB3*, *HOXB4*, *HOXB5*, *HOXC4*, *HOXD3*, *MEIS1*, *PBX1*, *NR2F1*, *STRA6*, and *RDH5*. These findings suggest the presence of an RA/*HOX*-associated developmental signature within the CoA transcriptomic profile. Cardiovascular and vascular signaling pathways were also represented, although they were less dominant than the ECM and developmental patterning signatures. These included pathways related to cardiac conduction, cardiac hypertrophy signaling, β-adrenergic signaling, PDGF signaling, and vasoactive or calcium-handling processes, involving molecules such as *EDNRA*, *PDGFB*, *PDGFRB*, *CACNB2*, *KCNA5*, *RYR2*, *SLC8A1*, *SLC8A2*, *PDE1C*, *PDE3B*, and *PDE5A*.

In summary, canonical pathway analysis highlighted a dominant ECM and collagen-remodeling signature together with developmental RA/*HOX*-associated annotations and cardiovascular signaling components within the CoA transcriptomic profile.

**Figure 3:**
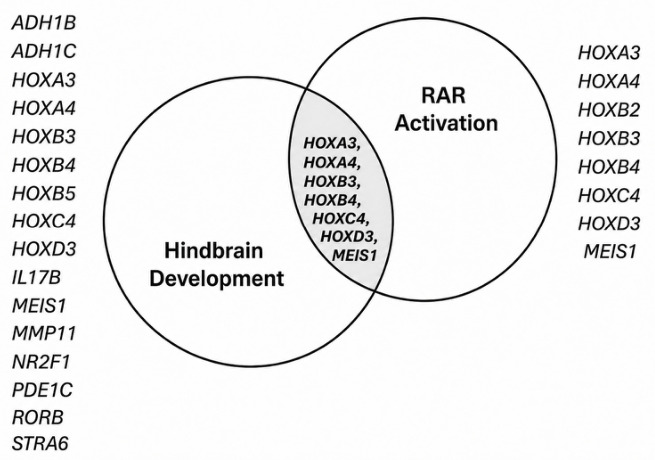
Shared RA/*HOX/MEIS*-associated developmental annotations identified by overlap analysis. Overlap analysis of IPA-derived developmental annotations identified a shared gene set linking RA/RAR-associated signaling with *HOX*-related developmental annotations.

To further characterize the developmental component within the canonical pathway results, overlap analysis was performed between enriched RA/RAR-associated and anterior *HOX*/hindbrain developmental annotations. The strongest overlap was observed between RAR activation and activation of anterior *HOX* genes in hindbrain during early embryogenesis (Figure 3). In this context, the hindbrain-related IPA annotation was interpreted as an embryonic anterior–posterior patterning program rather than as a brain-specific signal, because anterior *HOX* expression domains are closely linked to hindbrain segmentation, pharyngeal arch patterning, and developmental patterning processes relevant to pharyngeal arch and neural crest biology. Shared molecules included *HOXA3*, *HOXA4*, *HOXB3*, *HOXB4*, *HOXC4*, *HOXD3*, and *MEIS1*, suggesting that the observed developmental annotation was associated with a coherent *HOX*/*MEIS*-related gene set rather than by isolated pathway annotations. This shared signature is consistent with overlap between retinoid-associated developmental annotations and *HOX*/*MEIS*-related patterning programs within the CoA transcriptomic profile. Additional exploratory overlap analyses involving ductal- and neural crest-associated annotations identified candidate molecules such as *TFAP2B* and *EDNRA*. Because these annotations were not among the dominant canonical pathway results, they were interpreted as supportive exploratory findings and not as primary evidence for a neural crest-specific mechanism.

### 3.3. Network Analysis Highlights Remodeling and Developmental Patterning Modules

IPA regulator effect network analysis identified 25 networks. The complete network output, including the detailed list of associated molecules for each network, is provided in Supplementary Table S3. Because IPA network annotations are based on shared molecular components, they were interpreted as functional modules rather than disease-specific diagnoses. For biological interpretation, we focused on the highest-scoring and most coherent networks related to ECM remodeling, smooth muscle/cytoskeletal organization, and developmental patterning (Table 4).

Among the remodeling-associated networks, Networks 1, 7, and 8 were dominated by ECM, collagen, muscle, and cytoskeletal components. Network 1 was annotated to organ development, organ morphology, and skeletal and muscular system development and function, and included molecules related to contractile and cytoskeletal organization, including *CSRP1*, *CSRP2*, *HSPB2*, *MYOZ1*, *SORBS2*, *SYNPO2*, *TNNC1*, and *TNNT1*, as well as matrix- and growth-factor-associated molecules such as *MMP11*, *IGFBP3*, *IGFBP5*, and *TFPI2*. Networks 7 and 8 further reinforced the ECM signature, containing multiple collagen, basement membrane, and collagen-modifying components, including *COL13A1*, *COL4A5*, *COL8A1*, *COL11A2*, *COL4A6*, *COL6A6*, *COL8A2*, *COL9A1*, *PLOD2*, *LAMC3*, *HSPG2*, and *EMILIN1*.

In parallel, Networks 2 and 4 contained developmental patterning regulators. Network 2 was annotated to connective tissue disorders, embryonic development, and organismal development, and included multiple *HOX* genes and developmental cofactors, including *HOXA3*, *HOXA5*, *HOXB2*, *HOXB3*, *HOXB4*, *HOXB5*, *HOXB6*, *HOXC4*, *HOXD3*, *MEIS1*, and *PBX1*. Notably, this network also contained *STRA6*, *TAGLN*, *TEAD*, and *YAP*/*TAZ*, suggesting overlap between retinoid-associated developmental patterning, smooth muscle phenotype, and mechano-transductive regulation. Network 4 included *DLX1*, *DLX2*, *DLX5*, *DLX6*, *HAND2*, *HOXA4*, RAR/RXR/retinoic acid, *RDH5*, *GLI3*, *SOX11*, *WNT2B*, *FRZB*, and *SFRP4*, and was annotated to embryonic development, organismal development, and skeletal and muscular system development and function.

Together, these networks suggest that the CoA transcriptomic profile contains both a dominant matrix-remodeling component and a developmental patterning-associated component involving RA/RAR-, *HOX*/*MEIS*/*PBX*-, *DLX*/*HAND2*-, and TEAD/YAP-TAZ-related molecules.

### 3.4. Upstream Regulator Analysis Suggests Developmental, Epigenetic, and Vascular Remodeling Regulators

Upstream regulator analysis was interpreted as hypothesis-generating and used to contextualize the pathway- and network-level findings. The top upstream regulators included beta-estradiol, *MEF2C*, *KAT6A*, and the RAR/RXR/retinoic acid complex (Table 5), followed by additional chromatin-associated and developmental regulators, including *ARID1A*, *KAT6B*, *DNMT3B*, and *KMT2A*. The predicted activation of *KAT6A*, *KAT6B*, *KMT2A*, *ARID1A*, and RAR/RXR/retinoic acid, together with predicted inhibition of *DNMT3B* and *ACTR5*, was consistent with the *HOX*/*DLX*/*MEIS*/*PBX* developmental patterning signals identified in the canonical pathway and network analyses.

In parallel, inferred activation of *MEF2C*, *TGFB1*, *AGT*, and *MYO6* supported involvement of muscle, vascular, cytoskeletal, and matrix-remodeling programs. *MEF2C* was particularly consistent with the smooth muscle and mesenchymal regulatory modules identified in the network analysis, whereas *TGFB1* and *AGT* aligned with fibrosis-like remodeling and vascular signaling pathways.

Beta-estradiol showed the strongest *p*-value of overlap among the upstream regulators. Given the male infant CoA tissue context and the broad pleiotropic effects represented by estrogen-responsive IPA networks, this regulator was interpreted cautiously. Rather than indicating a primary estrogen-driven mechanism, the beta-estradiol-associated output was considered to reflect shared downstream programs involving proliferation, growth-factor responsiveness, ECM remodeling, and vascular or mesenchymal tissue adaptation.

Together, the upstream regulator results are consistent with a regulatory framework involving developmental/epigenetic patterning and smooth muscle/vessel-wall remodeling programs within CoA tissue. To further contextualize selected upstream regulator networks, cellular localization maps of target molecules associated with *MEF2C* and *KAT6A* are provided in (Supplementary Tables S4 and S5, respectively), illustrating their IPA-predicted distribution across nuclear, cytoplasmic, membrane-associated, and extracellular compartments.

### 3.5. Quantitative PCR Follow-Up of Selected Developmental and Vascular Candidate Genes

**Figure 4:**
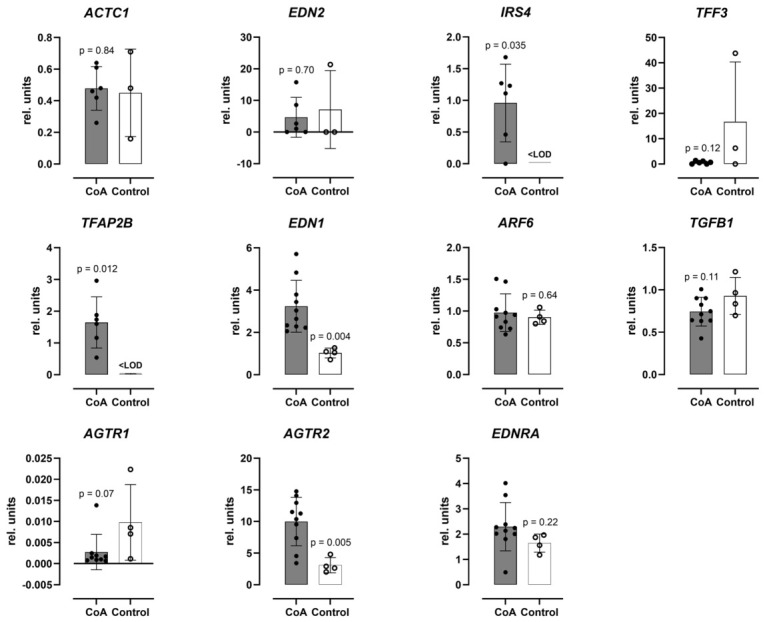
Relative mRNA expression of selected candidate genes in two independent cDNA sets from male CoA and Ao control samples. Relative quantitative PCR values were normalized to the housekeeping gene *HPRT1*. Bars indicate mean ± SD. Group comparisons were performed using Welch’s *t*-test. Significantly higher expression in CoA samples was observed for *EDN1*, *AGTR2*, *IRS4*, and *TFAP2B* (*p* < 0.05). Legend: CoA, coarctation of the aorta; Ao, ascending aortic control tissue; *HPRT1*, hypoxanthine phosphoribosyltransferase 1; SD, standard deviation; Ct, cycle threshold; LOD, lower limit of detection, defined as Ct ≥ 45.

To orthogonally validate selected array findings, relative quantitative PCR was performed in two independent cDNA sets from male CoA (n = 6; n = 8) and Ao control (n = 3; n = 4) samples (Figure 4). The analyzed targets included genes related to endothelin signaling, renin–angiotensin signaling, developmental regulation, growth-factor-associated signaling, and tissue remodeling. Candidate genes were selected to represent key biological modules emerging from the transcriptomic analysis, including endothelin and angiotensin-associated vascular signaling (*EDN1*, *EDN2*, *EDNRA*, *AGTR1*, *AGTR2*), developmental transcriptional regulation (*TFAP2B*), growth-factor/metabolic signaling (*IRS4*), cytoskeletal or remodeling-associated regulation (*ACTC1*, *ARF6*, *TGFB1*), and epithelial/tissue-repair-associated signaling (*TFF3*). Significantly higher relative expressions in CoA tissue were observed for endothelin 1 (*EDN1*) (*p* = 0.0002), angiotensin II receptor type 2 (*AGTR2*) (*p* = 0.0003), insulin receptor substrate 4 (*IRS4*) (*p* = 0.01), and transcription factor AP-2 beta (*TFAP2B*) (*p* = 0.004). Expression of *EDN2*, endothelin receptor type A (*EDNRA/ETA*), angiotensin II receptor type 1 (*AGTR1*), ADP-ribosylation factor 6 (*ARF6*), transforming growth factor beta 1 (*TGFB1*), actin alpha cardiac muscle 1 (*ACTC1*), and trefoil factor 3 (*TFF3*) did not differ significantly between CoA and Ao control tissue. Thus, qPCR validation confirmed differential expression of selected endothelin-, angiotensin-, *IRS4*-, and *TFAP2B*-associated candidate genes, while other array- or pathway-derived targets, including *TGFB1*, were not validated at the transcript level in the qPCR follow-up.

## 4. Discussion

### 4.1. Summary of Main Findings

This study identified transcriptomic signatures consistent with both developmental patterning and vessel-wall remodeling processes in human infantile CoA tissue. Canonical pathway analysis revealed a dominant ECM and collagen-remodeling profile, including collagen biosynthesis and degradation, integrin-mediated cell–matrix interaction, wound-healing signaling, and fibrosis-like remodeling. At the same time, the transcriptomic profile retained developmental features, including RAR/RXR signaling, anterior *HOX* patterning, and a shared *HOX*/*MEIS* signature. Network and upstream regulator analyses were consistent with this dual architecture, identifying ECM-, cytoskeletal-, and muscle-associated modules alongside developmental and epigenetic regulators including *KAT6A*, *KAT6B*, *RAR*/*RXR*/retinoic acid, *DNMT3B*, *KMT2A*, and *ARID1A*.

These findings support the hypothesis that developmental patterning-associated signatures coexist with postnatal vessel-wall remodeling processes in infantile CoA rather than representing a purely congenital defect or an isolated fibrotic process. Smooth muscle, mechano-transductive and vascular signaling pathways, including MEF2C-, TEAD/YAP-TAZ-, PDGF-, endothelin-, and angiotensin-associated signaling, may provide links between developmental patterning and vascular remodeling. RT-qPCR confirmed increased expression of *EDN1*, *AGTR2*, *IRS4*, and *TFAP2B* in CoA tissue, although not all pathway-derived targets, supporting selected vascular and developmental candidate genes.

**Figure 5:**
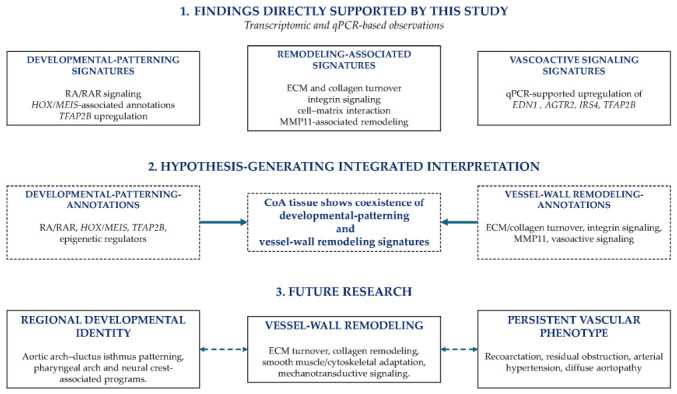
Developmental- and remodeling-associated signatures in infantile coarctation of the aorta: a hypothesis-generating framework. The figure summarizes the main molecular themes identified in the present study and separates directly supported findings from hypothesis-generating interpretation and future research directions. The upper section shows findings directly supported by transcriptomic and RT-qPCR analyses, including developmental-patterning signatures, extracellular matrix and vessel-wall remodeling-associated signatures, and qPCR-supported upregulation of selected vasoactive or developmental candidates. The middle section integrates these findings into a hypothesis-generating framework, proposing that infantile CoA tissue contains coexisting developmental-patterning-associated and vessel-wall remodeling-associated signatures. Dashed boxes indicate interpretative, hypothesis-generating elements rather than directly proven causal mechanisms. The lower section outlines future research areas suggested by this framework, including regional developmental identity of the aortic arch–ductus–isthmus region, mechanisms of vessel-wall remodeling, and persistent vascular phenotypes after anatomical repair. Abbreviations: CoA, coarctation of the aorta; RA, retinoic acid; RAR, retinoic acid receptor; *HOX*, homeobox; *MEIS*, myeloid ecotropic viral integration site 1 homolog; *TFAP2B*, transcription factor AP-2 beta; ECM, extracellular matrix; *MMP11*, matrix metalloproteinase 11; *EDN1*, endothelin 1; *AGTR2*, angiotensin II receptor type 2; *IRS4*, insulin receptor substrate 4; RT-qPCR, reverse-transcription quantitative polymerase chain reaction.

Overall, the data are consistent with a developmental-remodeling framework for infantile CoA, characterized by dominant ECM and collagen remodeling together with developmental transcriptional signatures involving RA/*HOX*-associated and *TFAP2B*-associated programs at the ductus–aortic isthmus interface. While this does not establish a primary neural crest-driven mechanism, it suggests that neural crest- and pharyngeal arch-associated developmental programs may contribute to the regional identity of the remodeled coarctation segment (Figure 5).

### 4.2. RA-HOX-Epigenetic Signatures as a Developmental Component of CoA Tissue

The identification of RAR/RXR signaling and anterior *HOX* developmental annotations is relevant to embryonic aortic arch and pharyngeal arch development. Retinoic acid is a key morphogen that regulates clustered *HOX* gene expression and contributes to anterior–posterior patterning during early embryogenesis [28,35]. Kashyap et al. [35] showed that retinoic acid induces coordinated transcriptional and epigenomic reorganization of *HOXA* and *HOXB* clusters, linking morphogen exposure to stable developmental gene regulation. In our dataset, RAR/RXR-associated signaling overlapped with anterior *HOX* developmental annotations through a shared *HOX*/*MEIS* gene set, supporting a coherent developmental annotation involving shared *HOX/MEIS*-associated molecules rather than isolated pathway enrichment. The IPA annotation referring to anterior *HOX* gene activation during hindbrain development should not be interpreted as a brain-specific signal in CoA tissue. Rather, it reflects an embryonic anterior–posterior patterning program. Hindbrain segmentation, pharyngeal arch patterning, and neural crest-associated regional identity are developmentally interconnected, and anterior *HOX* expression domains contribute to positional information within these embryonic territories [36]. Thus, the RA/*HOX*/*MEIS*-associated signature observed in CoA tissue is compatible with developmental patterning processes relevant to the aortic arch–ductal–isthmic region. This interpretation is supported by the concept that *HOX* expression can encode positional and developmental identity beyond embryogenesis. Hubert and Wellik [28] describe *HOX* genes as spatially and temporally regulated transcription factors that control embryonic patterning and may retain regionally restricted expression in postnatal and adult tissues. Trigueros-Motos et al. [29] demonstrated embryological-origin-dependent differences in homeobox expression in adult aortic segments, primarily in murine models, with additional validation in rat, pig, and human embryonic stem cell-derived smooth muscle cells. In that study, thoracic aorta-enriched *HOX* expression mainly involved *HOX6-10* paralogs, whereas the CoA-associated developmental signal in the present study involved more anterior *HOX* genes, including *HOXA3*, *HOXA4*, *HOXB3*, *HOXB4*, *HOXC4*, and *HOXD3*. This difference may reflect the distinct embryologic position of the aortic arch/isthmus region and supports the concept that regional *HOX* codes contribute to vessel-wall identity.

The vascular relevance of *HOX* programs is consistent with broader cardiovascular literature. Zhou et al. [30] summarize evidence that *HOX* genes participate in cardiac and vascular development, endothelial differentiation, vascular smooth muscle cell biology, vascular remodeling, and inflammatory vascular disease. Thus, the *HOX* signal observed in CoA tissue should not be viewed solely as an embryologic remnant. It may represent part of a developmental program influencing postnatal vascular phenotype and clinically relevant vessel-wall properties after repair, including arterial hypertension.

Although these findings cannot define cellular origin in bulk human CoA tissue, the presence of *HOX*-associated developmental annotations together with *TFAP2B* [37], RA/RAR signaling, *DLX/HAND2*-containing networks [38], and epigenetic regulators such as *KAT6A*, *KAT6B*, and *DNMT3B* is consistent with developmental programs that are known to participate in pharyngeal arch and neural crest biology [39,40].

### 4.3. TFAP2B at the Ductus–Aortic Isthmus Developmental Interface

This interpretation is supported by the anatomical localization of CoA. The aortic arch/isthmus region represents a developmental interface where embryonic patterning, ductal remodeling, neural crest-derived structures, and smooth-muscle lineage programs converge. *TFAP2B* is relevant in this context. Rothstein et al. [41] identified *TFAP2B* as part of a cranial neural crest gene regulatory program and showed that *TFAP2B* enhancer activity is controlled by developmental signaling inputs, including *WNT* and *SMAD2/3*-mediated TGF-β signaling. Although *TGFB1* expression was not confirmed as differentially expressed by RT-qPCR in our cohort, the role of *TFAP2B* within cranial neural crest regulatory circuits provides a biological rationale for interpreting increased *TFAP2B* expression in CoA tissue as part of a developmental transcriptional signature rather than as an isolated marker.

*TFAP2B* should not, however, be interpreted as a neural crest-specific marker alone. Germline mutations in *TFAP2B* cause Char syndrome, a familial heart–hand syndrome characterized by patent ductus arteriosus, facial dysmorphism, and hand anomalies [37]. *TFAP2B* therefore lies at the intersection of neural crest-associated developmental regulation, ductus arteriosus biology, and cardiovascular morphogenesis. This is particularly relevant for CoA given the anatomical proximity of the ductus arteriosus and aortic isthmus.

In the present dataset, *TFAP2B* occurred within a broader developmental-remodeling context that included RA/RAR signaling, anterior *HOX*-associated patterning, *DLX*/*HAND2*-containing networks, and ECM remodeling. *TFAP2B* should therefore be viewed not as proof of a neural crest-specific mechanism, but as one component of a broader developmental signature linking embryonic patterning, ductal/isthmic regional identity, and postnatal stenotic vessel-wall remodeling.

### 4.4. Extracellular Matrix Remodeling and Comparison with Previous Transcriptomic Studies

Although morphologic, histologic, and immunohistochemical features of CoA tissue have been described previously [42], molecular data from the localized coarctation segment remain limited. Ellegård et al. [14] performed transcriptomic profiling of pediatric CoA tissue and identified strong age- and sex-related effects, with younger patients showing increased ECM accumulation and fibrosis-related signaling. They also observed acute phase response, complement activation, and inflammatory pathway activation, including NF-κB-, MAPK-, and TGF-β-associated pathways.

Our study differs by using age- and sex-matched external control tissue and by focusing on male infants younger than one year, thereby reducing biological heterogeneity and accounting for the pronounced age- and sex-related transcriptional effects reported by Ellegård et al. [14]. Consistent with their study, we identified a strong ECM and collagen-remodeling signal. In contrast to their broader pediatric cohort, our controlled infant male cohort showed ECM remodeling together with developmental patterning programs, including RA/RAR signaling, anterior *HOX* developmental annotations, shared *HOX/MEIS*-associated molecules, *TFAP2B*, and epigenetic regulators such as *KAT6A*, *KAT6B*, and *DNMT3B*. The inflammatory signatures reported by Ellegård et al. [14] may additionally represent context-dependent downstream responses within an altered and remodeled vessel-wall environment rather than a competing mechanism. Thus, our findings extend prior inflammation- and remodeling-focused observations by placing ECM remodeling within a broader developmental-remodeling framework. The identification of MMP11- and collagen-related canonical pathway annotations further supports the presence of ECM-turnover-associated remodeling in CoA tissue. In heritable thoracic aortic diseases, increased circulating *MMP9* has been reported across Marfan syndrome, Loeys–Dietz syndrome, and familial thoracic aortic aneurysm associated with *ACTA2*, supporting altered ECM remodeling as a shared feature of genetic aortopathies [43]. Experimental work in fibrillin-1-deficient Marfan mice has further illustrated the functional relevance of the MMP/TIMP axis for aortic wall integrity [44,45,46]. Although these disorders represent aneurysmal rather than stenotic aortopathies, they demonstrate that dysregulated protease activity can substantially affect aortic wall architecture. MMP11 has been linked to ECM remodeling, tissue repair, organ development, and stromal microenvironment regulation, but its role in congenital aortic disease remains undefined [47]. In the present cohort, *MMP11* and collagen signals should therefore be interpreted as evidence of matrix remodeling accompanying the developmental transcriptional signature, not as evidence of a Marfan-like or aneurysmal mechanism.

### 4.5. Vascular Remodeling, Smooth Muscle Programs, and Clinical Implications

The present findings are clinically relevant because CoA is increasingly understood as a disease of the aortic wall rather than a purely discrete anatomical narrowing. Histologic and functional studies have described phenotypic modulation of smooth muscle cells, intimal thickening, impaired elastic fiber formation, altered collagen content, and reduced arterial elasticity in CoA [48]. Consistent with this concept, vascular abnormalities may persist after anatomically successful repair, including increased arterial stiffness, providing a biological context for recoarctation, residual arch obstruction, hypertension, and diffuse aortopathy despite relief of obstruction [49]. Current treatment recommendations also reflect this distinction between anatomical repair and long-term vascular disease. In neonates and young infants with native CoA, surgical resection of the narrowed segment remains the standard approach, whereas balloon angioplasty is generally associated with less durable relief and higher reintervention rates and is therefore generally limited to selected situations, such as temporary palliation when definitive repair or stent implantation is not feasible. In contrast, transcatheter treatment is particularly relevant for recurrent coarctation after surgical repair; in older children, adolescents, and adults with suitable anatomy, stent implantation is generally preferred because it provides structural support and more durable luminal stabilization [50]. In this context, our collagen- and *MMP*-related findings support a remodeling component within the coarctation segment. Network and upstream regulator analyses further implicated smooth muscle, cytoskeletal, mechano-transductive, and vascular signaling programs. *MEF2C*, TEAD/YAP-TAZ-associated network components, and *TEAD-SRF-MRTFB-MYOCD*-related regulator effects are consistent with smooth muscle and mesenchymal regulatory programs [51,52,53]. *PDGFB*/*PDGFRB*, *EDN1*/*EDNRA*, *AGT*/*AGTR2*, and calcium- or cyclic nucleotide-related molecules suggest altered vasoactive and remodeling-associated signaling [54,55,56]. RT-qPCR follow-up supported selected components of this vascular signaling layer, particularly increased *EDN1* and *AGTR2* expression, whereas *EDNRA*, *AGTR1*, and *TGFB1* were not significantly different. These findings support selected endothelin- and angiotensin-associated components of the proposed framework, but do not validate all pathway-derived candidates. 

Together, the persistence of developmental transcriptional and epigenetic regulatory programs, combined with vessel-wall remodeling signatures, may provide a biological rationale for structured long-term surveillance, morbidity reduction, and selected catheter-based reinterventions in patients with recurrent or residual obstruction. In this sense, developmental tissue biology may complement precision imaging and multidisciplinary follow-up by helping to frame CoA as a lifelong vascular condition rather than an isolated neonatal repair problem.

### 4.6. Limitations

Several limitations should be considered. First, the cohort was restricted to male infants younger than one year. This approach reduced biological heterogeneity and minimized the sex- and age-related transcriptional differences reported in previous studies; however, it limits generalizability to female patients and older age groups. Second, although control tissue was free of CoA, it was obtained from patients undergoing surgery for other cardiac conditions and may not fully represent normal aortic tissue. Furthermore, complete anatomical equivalence between CoA and control tissue cannot be guaranteed. CoA samples were obtained from the resected stenotic aortic isthmus segment, whereas control tissue was collected from macroscopically normal-appearing aortic tissue during surgery for other congenital cardiovascular malformations. Regional vascular expression differences, particularly involving developmental patterning genes, may therefore have contributed to the observed transcriptional profile. Previous studies have demonstrated region-specific *HOX* expression patterns along the normal aorta, particularly involving *HOX6-10* paralog groups that contribute to positional identity along the aortic axis [29]. Moreover, *HOX*-dependent developmental and neural crest patterning programs are highly conserved across vertebrate species [25]. However, the *HOX*-associated signature identified in the present study was dominated by different family members, including *HOXB2–HOXB5*. While anatomical variation may therefore have contributed to the observed developmental annotations, it is unlikely to fully explain the specific transcriptional signature observed in CoA tissue. Nevertheless, this possibility cannot be formally excluded.

The sample size was limited by the rarity of human infant CoA tissue samples and the restricted availability of suitable control tissue. This reduced statistical power and increased susceptibility to regional tissue heterogeneity within the aortic arch and isthmus.

The use of an exploratory FDR threshold of 0.20 reflects the limited availability of surgically resected infant aortic tissue and the resulting constraints on statistical power. Nevertheless, more than 200 transcripts remained significant at FDR < 0.05, supporting the robustness of the overall transcriptional signal despite the exploratory study design.

Because the present study was designed as a molecular analysis of archived surgically resected tissue rather than as a clinical outcome study, comprehensive longitudinal follow-up data, standardized pre- and postoperative blood pressure or hemodynamic measurements, perioperative laboratory parameters, and detailed postoperative imaging data were not available in a uniform manner and could not be analyzed systematically.

Additionally, direct comparison with other transcriptomic studies, including Ellegård et al. [14], is limited by differences in platform, analytical pipeline, tissue selection, control strategy, and access to raw datasets.

The transcriptomic and pathway-based analyses were exploratory. The IPA input gene set was derived using filtering criteria appropriate for hypothesis generation in a small-sample setting. Upstream regulator analysis reflects inference from known regulator–target relationships rather than direct evidence of regulator activity. IPA disease and function annotations should therefore be interpreted as functional modules based on shared molecular components, not as disease-specific diagnoses.

RT-qPCR was performed in independent cDNA sets rather than on the identical RNA specimens used for array profiling due to the small sample sizes available. These analyses should therefore be interpreted as candidate-gene follow-up rather than direct technical validation. Differences in tissue composition, platform-specific probe detection, transcript variants, and small sample size may have contributed to differences in expression direction or significance for individual genes. Accordingly, the pathway, network, and upstream regulator results should be regarded as hypothesis-generating rather than mechanistic proof. Transcript-level findings supported by RT-qPCR should therefore be distinguished from pathway-level hypotheses generated by downstream bioinformatic analyses.

Finally, the bulk-tissue approach cannot determine the cellular origin of the observed signatures. CoA tissue contains smooth muscle cells, endothelial cells, fibroblast-like cells, resident immune cells, and ECM-rich regions. Developmental patterning signatures, ECM remodeling programs, and vascular signaling changes therefore cannot yet be assigned to specific cell populations or anatomical subregions.

## 5. Outlook and Conclusions

Future studies should validate these findings in larger, sex-inclusive cohorts and apply spatially resolved approaches, including spatial transcriptomics, single-cell or single-nucleus RNA sequencing, and multiplex tissue imaging, to define the cellular and regional origin of the observed transcriptional signatures. Key questions include whether the *HOX*-associated developmental annotations observed in CoA tissue reflect regional developmental patterning, persistent tissue identity, remodeling-associated responses, or a combination thereof [30]. Given the enrichment of chromatin-associated regulators such as *KAT6A*, *KAT6B*, *DNMT3B*, *KMT2A*, and *ARID1A*, future epigenetic studies may further clarify how RA/*HOX*-associated developmental programs are established, maintained, or modified within the aortic arch–ductus–isthmus region.

In conclusion, infantile CoA tissue displayed a dominant ECM and collagen-remodeling signature accompanied by RA/*HOX*/*MEIS*-associated developmental annotations, smooth muscle/cytoskeletal regulatory programs, and selected vascular signaling changes. These findings are consistent with a developmental-remodeling framework for CoA in which developmental patterning-associated signatures coexist with postnatal vessel-wall remodeling. While the present data do not establish specific developmental mechanisms or cellular origins, they suggest that developmental and remodeling processes may jointly contribute to the molecular landscape of the coarctation segment and provide a hypothesis-generating framework for future studies of persistent vascular abnormalities beyond anatomical repair.

## Figures and Tables

**Table 1:** Overview of concomitant cardiovascular defects of the control group (Ao). Legend: ASD, atrial septal defect; VSD, ventricular septal defect; d-TGA, dextro-transposition of the great arteries; PA, pulmonary atresia; MAPCA, major aortopulmonary collateral arteries; PS sub, subvalvular pulmonary stenosis; PFO, patent foramen ovale; CAA, coronary artery anomaly.

Major Defect(s)	Minor Defect(s)
d-TGA	ASD II
d-TGA	MI
d-TGA	ASD II, VSD
PA + VSD + MAPCA	PFO
PS sub	VSD, ASD, CAA
d-TGA	ASD

**Table 2:** Transcripts with the largest absolute expression differences in CoA tissue compared with control vascular tissue. Transcripts subsequently assessed by qPCR are highlighted in bold. Legend: CoA, coarctation of the aorta; qPCR, quantitative polymerase chain reaction.

	Name	Entrez Gene	Log Ratio	*p*-Value	Molecule Type
Up-regulated	*TFAP2B*	transcription factor AP-2 beta	7.03	4 × 10^−16^	transcription regulator
	*IRS4*	insulin receptor substrate 4	6.88	2 × 10^−16^	other
	*KRT14*	keratin 14	6.07	4 × 10^−12^	other
	*CBLN2*	cerebellin 2 precursor	5.78	2 × 10^−10^	other
	*DLX2*	distal-less homeobox 2	5.37	2 × 10^−14^	transcription regulator
	*KLK5*	kallikrein related peptidase 5	5.04	2 × 10^−10^	peptidase
	*FOXD1*	forkhead box D1	4.86	9 × 10^−11^	transcription regulator
	*HOXD3*	homeobox D3	4.68	1 × 10^−11^	transcription regulator
	*FOXF1*	forkhead box F1	4.67	1 × 10^−10^	transcription regulator
	*DLX1*	distal-less homeobox 1	4.59	4 × 10^−11^	transcription regulator
	*KRT17*	keratin 17	4.59	6 × 10^−10^	other
	*SLITRK6*	SLIT and NTRK like family member 6	4.58	2 × 10^−8^	other
	*PDE1C*	phosphodiesterase 1C	4.54	9 × 10^−9^	enzyme
	*GRIK1*	glutamate ionotropic receptor kainate type subunit 1	4.28	1 × 10^−8^	ion channel
	*MAB21L2*	mab-21 like 2	4.05	8 × 10^−9^	other
	**Name**	**Entrez Gene**	**Log Ratio**	***p*** **-Value**	**Molecule Type**
Down-regulated	*HLA-DQB1*	major histocompatibility complex, class II, DQ beta 1	−3.2	0.0004	other
	*C7*	complement C7	−3.14	3 × 10^−6^	other
	*TFPI2*	tissue factor pathway inhibitor 2	−3.03	8 × 10^−7^	other
	*CCL21*	C-C motif chemokine ligand 21	−3	0.0004	cytokine
	*MMRN1*	multimerin 1	−2.98	2 × 10^−6^	other
	*IRX2*	iroquois homeobox 2	−2.83	6 × 10^−6^	transcription regulator
	*NPY*	neuropeptide Y	−2.8	6 × 10^−5^	other
	*LAMC3*	laminin subunit gamma 3	−2.79	3 × 10^−5^	other
	*TSPAN8*	tetraspanin 8	−2.73	7 × 10^−6^	other
	*ADRA1B*	adrenoceptor alpha 1B	−2.69	4 × 10^−6^	G-protein-coupled receptor
	*TFF3*	trefoil factor 3	−2.69	0.0029	other
	*RIMS2*	regulating synaptic membrane exocytosis 2	−2.67	2 × 10^−5^	other
	*SERPINA3*	serpin family A member 3	−2.5	0.001	other
	*GATA5*	GATA binding protein 5	−2.35	4 × 10^−5^	transcription regulator
	*CRLF1*	cytokine receptor like factor 1	−2.23	0.0009	cytokine

**Table 3:** Enriched canonical pathways identified by IPA. Pathway names are shown as provided by IPA. Disease-specific pathway labels, such as hepatic or pulmonary fibrosis, were interpreted as indicating shared extracellular matrix and fibrosis-like remodeling programs rather than organ-specific liver or lung pathology. Canonical pathways are ranked by −log(*p* value). The z-score indicates predicted pathway activation or inhibition where available and was not used as the primary ranking criterion. Legend: IPA, Ingenuity Pathway Analysis; NA, not available.

	−log(*p*-Value)	Ratio	z-Score
Hepatic Fibrosis/Hepatic Stellate Cell Activation	8.88	0.0885	NA
Collagen biosynthesis and modifying enzymes	8.78	0.164	2.714
Pulmonary Fibrosis Idiopathic Signaling Pathway	8.30	0.0644	4.025
Collagen chain trimerization	8.17	0.205	2.333
Collagen degradation	7.82	0.156	2.530
Integrin cell surface interactions	7.66	0.129	2.714
RAR Activation	6.79	0.0506	2.236
Wound Healing Signaling Pathway	6.45	0.0643	3.500
GP6 Signaling Pathway	5.83	0.0859	1.897
Assembly of collagen fibrils and other multimeric structures	5.77	0.131	2.121
Activation of anterior *HOX* genes in hindbrain during early embryogenesis	5.20	0.0826	3.162
Extracellular matrix organization	4.80	0.0841	1.667
Pathogen Induced Cytokine Storm Signaling Pathway	4.66	0.0445	2.673
Role of Osteoclasts in Rheumatoid Arthritis Signaling Pathway	4.49	0.0475	1.807
Cardiac conduction	4.13	0.0692	2.333

**Table 4:** Top 15 IPA regulator effect networks ranked by IPA network score. The table shows the highest-ranking networks identified by IPA regulator effect network analysis, including network score, number of focus molecules, and IPA-assigned top diseases and functions. The complete network output, including all 25 networks and the associated molecules for each network, is provided in Supplementary Table S3. IPA-assigned disease and function terms were interpreted as functional annotations based on shared molecular components rather than as evidence of disease-specific diagnoses. Legend: IPA, Ingenuity Pathway Analysis.

#	Score	Focus Molecules	Top Diseases and Functions
1	43	24	[Organ Development, Organ Morphology, Skeletal and Muscular System Development and Function]
2	43	24	[Connective Tissue Disorders, Embryonic Development, Organismal Development]
3	43	24	[Cell Death and Survival, Gene Expression, Organismal Injury and Abnormalities]
4	40	23	[Embryonic Development, Organismal Development, Skeletal and Muscular System Development and Function]
5	31	19	[Cellular Development, Cellular Function and Maintenance, Protein Synthesis]
6	29	18	[Endocrine System Disorders, Gastrointestinal Disease, Hereditary Disorder]
7	27	17	[Connective Tissue Disorders, Dermatological Diseases and Conditions, Organismal Injury and Abnormalities]
8	25	16	[Connective Tissue Disorders, Dermatological Diseases and Conditions, Organismal Injury and Abnormalities]
9	25	16	[Cell Signaling, Cellular Function and Maintenance, Vitamin and Mineral Metabolism]
10	25	16	[Behavior, Cell-To-Cell Signaling and Interaction, Digestive System Development and Function]
11	23	15	[Cardiovascular Disease, Organismal Injury and Abnormalities, Skeletal and Muscular Disorders]
12	23	15	[Cancer, Organismal Injury and Abnormalities, Skeletal and Muscular Disorders]
13	23	15	[Behavior, Cancer, Nervous System Development and Function]
14	23	15	[Endocrine System Disorders, Gastrointestinal Disease, Inflammatory Disease]
15	23	15	[Cellular Assembly and Organization, Hair and Skin Development and Function, Molecular Transport]

**Table 5:** Top upstream regulators identified by IPA upstream regulator analysis. Regulators are ranked by *p*-value of overlap. Predicted activation state and activation z-score are shown where available. Upstream regulator analysis was interpreted as hypothesis-generating and used to contextualize the canonical pathway and network findings. The complete upstream regulator output is provided in Supplementary Table S4. Legend: NA, not available; A, predicted activation; I, predicted inhibition; IPA, Ingenuity Pathway Analysis.

Upstream Regulator	Predicted Activation State	Activation z-Score	*p*-Value of Overlap	Molecule Type
beta-estradiol	A	2.195	9.08 × 10^−21^	chemical—endogenous mammalian
*MEF2C*	A	2.941	8.98 × 10^−15^	transcription regulator
*KAT6A*	A	3.41	5.76 × 10^−13^	enzyme
RAR/RXR/RETINOIC ACID (complex)	A	2.714	7.15 × 10^−11^	complex
*LMNA*	NA	0.743	7.82 × 10^−11^	other
*TP63*	NA	0.154	1.04 × 10^−10^	transcription regulator
*TGFB1*	A	2.465	2.46 × 10^−10^	growth factor
*ACTR5*	I	−2.496	5.43 × 10^−10^	other
*ARID1A*	A	2.2	6.52 × 10^−10^	transcription regulator
*MYO6*	A	2.309	1.19 × 10^−9^	other
*THRA*	NA	1.953	2.12 × 10^−9^	ligand-dependent nuclear receptor
*KAT6B*	A	2.828	2.19 × 10^−9^	enzyme
*AGT*	A	2.832	2.95 × 10^−9^	growth factor
*HNRNPA2B1*	NA	NA	4.09 × 10^−9^	other
*ESR2*	NA	0.69	4.14 × 10^−9^	ligand-dependent nuclear receptor
*DNMT3B*	I	−2.309	6.85 × 10^−9^	enzyme
*CREB1*	NA	1.444	8.98 × 10^−9^	transcription regulator
*KMT2A*	A	2.932	1.16 × 10^−8^	enzyme

## Data Availability

The gene expression data supporting the findings of this study have been deposited in NCBI’s Gene Expression Omnibus (GEO) and are accessible under GEO Series accession number GSE337213. Additional data supporting the findings of this study are available from the corresponding author upon reasonable request.

## Supplementary Materials

The following supporting information can be downloaded at: https://www.mdpi.com/article/10.3390/jcm15135214/s1, Table S1: Primer sequences for quantitative real time PCR analysis; Table S2: IPA canonical pathways with pathway-associated molecules; Table S3: IPA Networks including associated molecules for each network; Table S4: Upstream Regulators, *MEF2C*-associated molecules; Table S5: Upstream Regulators, *KAT6A*-associated molecules. Note: Supplementary IPA output files are provided in their original software-generated format. Accordingly, gene symbols are not italicized in these exported tables.

## Abbreviations

The following abbreviations are used in this manuscript:

Ao	Ascending Aorta Control Tissue
CoA	Coarctation of the Aorta
Ct	Cycle Threshold
d-TGA	Dextro-Transposition of the Great Arteries
ECM	Extracellular Matrix
FDR	False Discovery Rate
GEO	Gene Expression Omnibus
IPA	Ingenuity Pathway Analysis
logFC	Log2 Fold Change
MAPCA	Major Aortopulmonary Collateral Artery
PDA	Patent Ductus Arteriosus
PFO	Patent Foramen Ovale
PS sub	Subvalvular Pulmonary Stenosis
RA	Retinoic Acid
RAR	Retinoic Acid Receptor
RIN	RNA Integrity Number
RNA	Ribonucleic Acid
RQ	Relative Quantitation
RT-qPCR	Reverse Transcription Quantitative Polymerase Chain Reaction
RXR	Retinoid X Receptor
SD	Standard Deviation
TGF-β	Transforming Growth Factor Beta
UMAP	Uniform Manifold Approximation and Projection
VSD	Ventricular Septal Defect
