# Supplementary material for: Developmental and Structural Alterations at the Ductus–Aortic Isthmus Interface in Infantile Coarctation of the Aorta: A Biological Basis for Persistent Vascular Disease Beyond Anatomical Repair
Source: J Clin Med. 2026 Jul 3;15(13):5214. doi: 10.3390/jcm15135214 (PMC13362620; doi:10.3390/jcm15135214)
Supplement: Supplementary file 1 [file jcm-15-05214-s001.zip › Table S1.pdf]

Supplementary Table 1. Primer sequences for quantitative real time PCR analysis.

Gene	Primer	Nucleotide sequence
ACTB	Taq	5' CCA GCC ATG TAC GTT GCT ATC CAG GC 3'
	Forw	5' CCG CGA GAA GAT GAC CCA G 3'
	Rev	5' CCA GTG GTA CGG CCA GAG G 3'
GAPDH	Taq	5' CCT CAA CTA CAT GGT TTA CAT GTT CCA ATA T 3'
	Forw	5' GCC ATC AAT GAC CCC TTC ATT 3'
	Rev	5' TTG ACG GTG CCA TGG AAT TT 3'
HPRT1	Taq	5' CGC AGC CCT GGC GTC GTG ATT A 3'
	Forw	5' CCG GCT CCG TTA TGG C 3'
	Rev	5' GGT CAT AAC CTG GTT CAT CAT CA 3'
EDN1	Taq	5' TTC TGC CAC CTG GAC ATC ATT TGG G 3'
	Forw	5' CTG CTC GTC CCT GAT GGA TAA 3'
	Rev	5' GGC TTC CAA GTC CAT ACG GA 3'
EDN2	Taq	5' GGG CCA CTA CAG GAG AGC TT 3'
	Forw	5' TGT TCC AGA CTG GCA AGA CA 3'
	Rev	5' GAA ATG TCC CTC AGC CTT TG 3'
TFF3	Taq	5' GCT GCT TTG ACT CCA GG ATC 3'
	Forw	5' CAT GTC ACC CCC AAG GAG T 3'
	Rev	5' AAG GTG CAT TCT GCT TCC TG 3'
TFAP2B	Taq	5' TCC CAA ATC GGT GAC TTC TC 3'
	Forw	5' GCG GCA TGA ATC TAT TGG AC 3'
	Rev	5' CCC AGG AAG CCG TCT TTA TT 3'
IRS4	Taq	5' GAC GAA TAC TTC GCG ATG GT 3'
	Forw	5' GGT ACC GAC ACC TCA TTG CT 3'
	Rev	5' GCA GCA AGT ACC AGC TTT CC 3'
ACTC1	Taq	5' ACG TGA AAT TGT CCG TGA CA 3'
	Forw	5' CTG AGC GTG GCT ACT CCT TT 3'
	Rev	5' AGC TGT GGC CAT CTC ATT CT 3'
TGFB1	Taq	5' CGA GGT GAC CTG GCC ACC ATT CAT 3'
	ForW	5' GAC ATC AAC GGG TTC ACT A 3'
	Rev	5' CAT GAG AAG CAG GAA AGG CC 3'
AGTR1	Taq	5' CGA CGC ACA ATG CTT GTA GCC AAA GTC A 3'
	Forw	5' ACC CAA TGA AGT CCC GCC T 3'
	Rev	5' AGC AGC CAA ATG ATG ATG CAG 3'
AGTR2	Taq	5' AAC ATT ACC AGC GGT CTT CAC TTC GGG 3'
	Forw	5' CCA CCC TTG CCA CTA CTA GCA 3'
	Rev	5' ATT GTT GCC AGA GAT GTT CAC AA 3'
ARF6	Taq	5' CAG GAC CTG CCC GAT GCC ATG 3'
	Forw	5' AGG GAC GCC ATA ATC CTC ATC 3'
	Rev	5' GCC CAG TTT CTC CTG GAT CTC 3'
EDNRA	Taq	5' CCC TTG GTG TGC ACT GCG ATC TTC T 3'
	Forw	5' CAT GGA GTT CTA CCA AGA TGT AAA GG 3'
	Rev	5' CCT TCT GTT CAA CAT CTC ACA AGT C 3'
